# Novel Naturally Occurring Mutations of Enterovirus 71 Associated With Disease Severity

**DOI:** 10.3389/fmicb.2020.610568

**Published:** 2021-01-13

**Authors:** Chih-Shin Chang, Chun-Che Liao, An-Ting Liou, Yi-Chun Chou, Ya-Yen Yu, Chi-Yung Lin, Jen-Shiou Lin, Ching-Shu Suen, Ming-Jing Hwang, Chiaho Shih

**Affiliations:** ^1^Genomics Research Center, Academia Sinica, Taipei, Taiwan; ^2^Institute of Biomedical Sciences, Academia Sinica, Taipei, Taiwan; ^3^Graduate Institute of Medicine, Kaohsiung Medical University, Kaohsiung, Taiwan; ^4^Drug Development and Value Creation Research Center, Kaohsiung Medical University, Kaohsiung, Taiwan; ^5^Section of Clinical Virology and Molecular Diagnosis, Department of Laboratory Medicine, Changhua Christian Hospital, Changhua, Taiwan

**Keywords:** enterovirus 71, EV-A71, disease severity, mutations, VP1, VP2, SCARB2, PSGL-1

## Abstract

Infection with the re-emerging enterovirus 71 (EV-A71) is associated with a wide range of disease severity, including herpangina, encephalitis, and cardiopulmonary failure. At present, there is no FDA-approved therapeutics for EV-A71. Early diagnosis for the high-risk children is the key to successful patient care. We examined viral genome sequences at the 5′ untranslated region (UTR) and the capsid protein VP1 from 36 mild and 27 severe cases. We identified five EV-A71 mutations associated with severe diseases, including (1) the 5′ UTR mutations C580U, A707G, C709U; (2) a VP1 alanine-to-threonine mutation at position 280 (280T), and (3) a VP1 glutamic acid-to-(non-glutamic acid) at position 145 [145(non-E)]. SCARB2 is a known entry receptor for EV-A71. Based on a recent cryoEM structure of the EV-A71-SCARB2 binding complex, VP1-280T is near the binding interface between the VP1-VP2 complex and its entry receptor SCARB2. A *de novo* created hydrogen bonding between the mutant VP1-280T and the VP2-139T, could help strengthen a web-like interaction structure of the VP1-VP2 complex. A stabilized loop turn of VP2, once in contact with SCARB2, can enhance interaction with the host SCARB2 receptor for viral entry. Our findings here could facilitate early detection of severe cases infected with EV-A71 in clinical medicine. In addition, it opens up the opportunity of functional studies via infectious cDNA cloning, site-directed mutagenesis, and animal models in the future.

## Introduction

Reemergence of non-polio enterovirus is a new threat to the children (Ho et al., 1999; McMinn, 2002; Cox et al., 2017; Shih et al., 2018). Enterovirus 71 (EV-A71) is closely related to coxsackievirus, poliovirus, and hepatitis A virus (Racaniello, 2006; Feng et al., 2013; Sin et al., 2015). In a recent outbreak of EV-A71 in Shanghai, China, near 1000 deaths of children were reported (Zeng et al., 2012). Infection with EV-A71 is associated with a wide range of disease severity. In mild cases, hand-foot-and-mouth disease (HFMD) is common. In severe cases, it can lead to encephalitis, acute flaccid paralysis, tachycardia, cardiopulmonary failure, and death. Studies in various animal models demonstrated that EV-A71 can target multiple tissues, including the central nerve system (Cox et al., 2017; Shih et al., 2018), and recently, the cardiopulmonary system (Chang et al., 2019). Current treatment for EV-A71 remains supportive, and no antivirals are commercially available.

The broad spectrum of disease manifestations is related in part to the differences in host immunity (Liao et al., 2014; Liou et al., 2016, 2019). In addition, it has also been an active research topic whether sequence variation of EV-A71 could contribute to the different degrees and tropisms of pathogenesis *in vivo*. As summarized in Table 1, previous studies reported various viral mutations associated with disease severity in different human cases or mouse models. For example, amino acid 145E of VP1 is the most well documented mutation important for viral replication and pathogenesis in mouse models (Chua et al., 2008; Huang et al., 2012; Chang et al., 2018; Kobayashi et al., 2018; Tee et al., 2019). Similarly, in a monkey model, VP1-145E viruses can induce neurological symptoms, and it was suggested that VP1-145E has a replication advantage for the virus in the monkeys (Fujii et al., 2018).

**TABLE 1:** A summary of EV-A71 disease-associated mutations.

**Positions of mutation sites**			**Location on EV71 genome**	**Substitution mutations**				**Virus genotype**	**Patients and mouse samples**	**References**
				**Nucleotide**		**Amino acid**				
**a.a. numbering in mature viral protein**	**a.a. numbering in precursor polyprotein**	**Nucleotide number**		**Severe**	**Mild**	**Severe**	**Mild**			
		580	5′UTR	U	C			B5	63 (27 severe vs. 36 mild)	Our own data
		707	5′UTR	G	A			B5	63 (27 severe vs. 36 mild)	
		709	5′UTR	U	C			B5	63 (27 severe vs. 36 mild)	
VP1 145			VP1				Glu (E)	B5	63 (27 severe vs. 36 mild)	
VP1 280			VP1			Thr (T)	Ala (A)	B5	63 (27 severe vs. 36 mild)	
		150	5′UTR	G	A,U			C4 and B5	18 (9 severe vs. 9 mild)	Chang et al., 2012
		158	5′UTR	C	U			B1 and C2	2 (ICR mice)*	Yeh et al., 2011
		241	5′UTR	C	U			C4a	6 (1 severe vs. 5 mild)	Wen et al., 2013
		272	5′UTR	G				C4	56 (25 severe vs. 31 mild)	Li et al., 2011
		488	5′UTR	U				C4	56 (25 severe vs. 31 mild)	Li et al., 2011
		494	5′UTR	U	C			C2	1 (hSCARB2-Tg mice)*	Chang et al., 2018
		571	5′UTR	A	U			C4a	6 (1 severe vs. 5 mild)	Wen et al., 2013
		579	5′UTR	C	U			C4a	6 (1 severe vs. 5 mild)	Wen et al., 2013
		606	5′UTR	G	A			C4 and B5	18 (9 severe vs. 9 mild)	Chang et al., 2012
		700	5′UTR	A,U				C4	56 (25 severe vs. 31 mild)	Li et al., 2011
VP2 149			VP2	U	A	Met(M)	Lys(K)	C2 (mouse adaptive strain)	1 (ICR mice and Neuro-2a cell)*	Huang et al., 2012
VP2 149			VP2			Ile(I)	Lys(K)	B3 (mouse adaptive strain)	1 (BALB/c mice and CHO) cells)*	Chua et al., 2008
VP2 149			VP2			Met(M) Ile(I)	Lys(K)	B3, B4, C1, C2, and C4	5 (PSGL1-expressing L929 cell)*	Miyamura et al., 2011
VP2 149			VP2			Ile(I)	Lys(K)	B4	1 (ICR mice)*	Tee et al., 2019
VP1 97	P662		VP1	G	U	Arg(R)	Leu(L)	C	1 (SH-SY5Y cell)*	Cordey et al., 2012
VP1 104			VP1	G	A	Asp(D)	Asn(N)	C2	1 (hSCARB2-Tg mice)*	Chang et al., 2018
VP1 145			VP1			Gln(Q)	Glu(E)	C4 and B5	18 (9 severe vs. 9 mild)	Chang et al., 2012
VP1 145	P710		VP1			Gly(G) Gln(Q) Arg(R)	Glu(E)	C4	56 (25 severe vs. 31 mild)	Li et al., 2011
VP1 145			VP1			Glu(E)	Gln(Q) Gly(G)	C2	1 (hSCARB2-Tg mice)*	Chang et al., 2018
VP1 145			VP1	G	C	Glu(E)	Gln(Q)	C2 (mouse adaptive strain)	1 (ICR mice and Neuro-2a cell)*	Huang et al., 2012
VP1 145			VP1			Glu(E)	Gly(G)	B3 (mouse adaptive strain)	1 (BALB/c mice)*	Chua et al., 2008
VP1 145			VP1			Glu(E)	Gly(G)	B4 and C2	2 (cynomolgus macaque)	Fujii et al., 2018
VP1 145			VP1			Glu(E)	Gly(G)	B2, B4, and C2	3 (hSCARB2-Tg mice)*	Kobayashi et al., 2018
VP1 145			VP1			Glu(E)	Gln(Q)	B4	1 (ICR mice)*	Tee et al., 2019
VP1 146			VP1			Val(V)	Ile(I)	C2	1 (hSCARB2-Tg mice)*	Chang et al., 2018
VP1 164	P729		VP1			Glu(E)	Asp(D)	C4	56 (25 severe vs. 31 mild)	Li et al., 2011
VP1 170			VP1			Val(V)	Ala(A)	C2	10 (7 severe vs. 3 mild)	McMinn et al., 2001
VP1 241			VP1			Ser(S)	Leu(L)	C2	1 (hSCARB2-Tg mice)*	Chang et al., 2018
VP1 244			VP1			Glu(E)	Lys(K)	B4	1 (ICR mice)*	Tee et al., 2019
	P814		VP1			Val(V)	Ile(I)	C4a	31 (16 severe vs. 15 mild)	Wen et al., 2013
2A 68	P930		2A			Lys(K)	Met(M) Arg(R)	C4	56 (25 severe vs. 31 mild)	Li et al., 2011
	P1148		3A			Val(V)	Ile(I)	C4a	31 (16 severe vs. 15 mild)	Wen et al., 2013
	P1728		3C			Ala(A)	Cys(C) Val(V)	C4a	31 (16 severe vs. 15 mild)	Wen et al., 2013
3D 251			3D			Ile(I)	Thr(T)	C2	1 (ICR mice and SK-N-SH cell)*	Kung et al., 2010
	P1994		3D			Val(V)	Ile(I)	C4	6 (3 severe vs. 3 mild)	Chang et al., 2010
		7335	3′UTR	U	C			C4a	6 (1 severe vs. 5 mild)	Wen et al., 2013

*Indicates that these studies were based on cell lines or mouse samples.

In contrast to both mouse and monkey models, VP1-145E does not appear to be associated with severe sequelae using human samples. Instead, amino acids 145Q had been observed in 2 out of 9 severe diseases (Chang et al., 2012). In addition, 145Q, 145G, and 145R had been associated with virulent phenotype by analyzing 25 severe cases and 31 mild cases from the GenBank database (Li et al., 2011). It has remained a discrepancy between mouse models and human samples whether VP1-145E and disease severity are associated with each other. Amino acid 145 of VP1 was thought to be involved in the binding between EV-A71 and its entry receptor SCARB2 (scavenger receptor class B, member 2) (Victorio et al., 2016; van der Sanden et al., 2018). More recently, VP1-145 was shown to be related to binding to heparan sulfate (Tan et al., 2017; Fujii et al., 2018; Kobayashi et al., 2018, 2020). In this study using human clinical samples, we found five severity-associated mutations, including the 5′ UTR C580U, A707G, C709U, VP1-145(non-E), and VP1-280T. Interestingly, VP1-280T is located near the binding site with the VP2-EF loop and human SCARB2 receptor for viral entry (Zhou et al., 2019). Overall, our current results warrants further studies on the functional significance of these disease-associated mutations in animal models (Liao et al., 2014; Shih et al., 2018; Chang et al., 2019; Liou et al., 2019), once infectious cDNA clones can be successfully isolated from these patients in the future. Furthermore, our findings here could facilitate earlier detection based on the risk score in predicting severe cases infected with EV-A71 in clinical medicine. Hopefully, these novel mutations associated with disease severity could have a diagnostic potential in future epidemics of EV-A71.

## Materials and Methods

### Ethics Statement

EV-A71 clinical isolates were kindly provided by Section of Clinical Virology and Molecular Diagnosis, Department of Laboratory Medicine, Changhua Christian Hospital, Taiwan. Biosafety Committee approval number BSF 005 20080030 from Academia Sinica, Taiwan.

### Disease Severity

Clinical presentations of mild cases include herpangina or HFMD only. In addition to herpangina and HFMD, symptoms of severe cases include myoclonic jerks, meningitis, encephalitis, acute flaccid paralysis, pulmonary edema, cardiopulmonary failure, and death.

### Preparations of Cells and Viruses

Human rhabdomyosarcoma (RD) cells (ATCC CCL-136) were cultured in DMEM (Dulbecco’s modified Eagle medium) with 10% fetal bovine serum (FBS; HyClone) and 1% penicillin-streptomycin. Viral strains of EV-A71 were isolated from throat swabs of patients by the Section of Clinical Virology and Molecular Diagnosis, Department of Laboratory Medicine, Changhua Christian Hospital, Taiwan. Virus preparation was as described previously (Liao et al., 2014; Liou et al., 2016, 2019; Chang et al., 2019).

### RT-PCR and Sequencing

Procedures for RT-PCR are as described elsewhere (Liao et al., 2014). The sequencing strategy and primer sequences are shown in Supplementary Figure 3A. 5′ UTR and VP1 fragments were amplified using PCR primer pairs F1 and R4, F5 and R10, respectively. PCR-amplified DNA fragments were sequenced using the sequencing primers listed in Supplementary Figure 3B with ABI 3730XL DNA analyzer (PE Applied Biosystems, Foster City, CA, United States). Multiple sequence alignment was performed using the Clustal W multiple alignment program of the MegAlign software version 7.1.0 (DNASTAR, Madison, WI, United States).

### Binding Assay

SH-SY5Y, HTB-10 and RD cells were seeded in 6 well plate (1 × 10^6^/well) and cultured with Dulbecco’s Modified Eagle medium (DMEM; Gibco) with 10% fetal bovine serum (FBS; HyClone) and 1% penicillin-streptomycin (Gibco) at 37°C overnight. Culture medium was removed and cells were washed once with phosphate buffer saline (PBS) before incubation with EV-A71 virus VP1-280A or VP1-280T (M.O.I = 10) at 4°C for 1 h. Unbound virus was removed by washing with PBS three times. Total RNAs of cells were extracted with a WelPrep cell/tissue RNA kit (Wel-GENE), and were used for reverse transcription by a High-Capacity cDNA reverse transcription kit (Applied Biosystems). The synthetic cDNA was subjected to real-time quantitative PCR (qPCR) analysis by an ABI 7500 system with a Power SYBR green PCR master kit (both from Applied Biosystems). Specific primers for VP1 were CTAGAGGGTACCACCAATCC (forward) and AACCTGGCCAGTAGGAGT (reverse). The primer sequences of GAPDH (glyceraldehyde-3-phosphate dehydrogenase) were used as the internal control: ACCCAGAAGACTGTGGATGG (forward) and TCAGCTCAGGGATGACCTTG (reverse). The amounts of viral RNA were normalized to the levels of GAPDH.

### Bioinformatics

Discovery Studio Visualizer is from Dassault Systèmes BIOVIA Corp (2015), San Diego, CA United States. The PyMOL software is from PyMOL Molecular Graphics System, Version 1.8 Schrödinger, LLC.

### Statistical Analysis

Relative EV-A71 binding assay were analyzed by Student’s *t*-test. Mutation frequencies of viral genome were analyzed by the Chi-square test and the Fisher’s Exact test. ^∗^*P* < 0.05; ^∗∗^*P* < 0.001.

### GenBank Accession Numbers

VP1 sequences: MT348284 – MT348346; 5′ UTR sequences: MT360921 – MT360983; full-length EV-A71 genome: MT360984 – MT360998.

## Results

**FIGURE 1:**
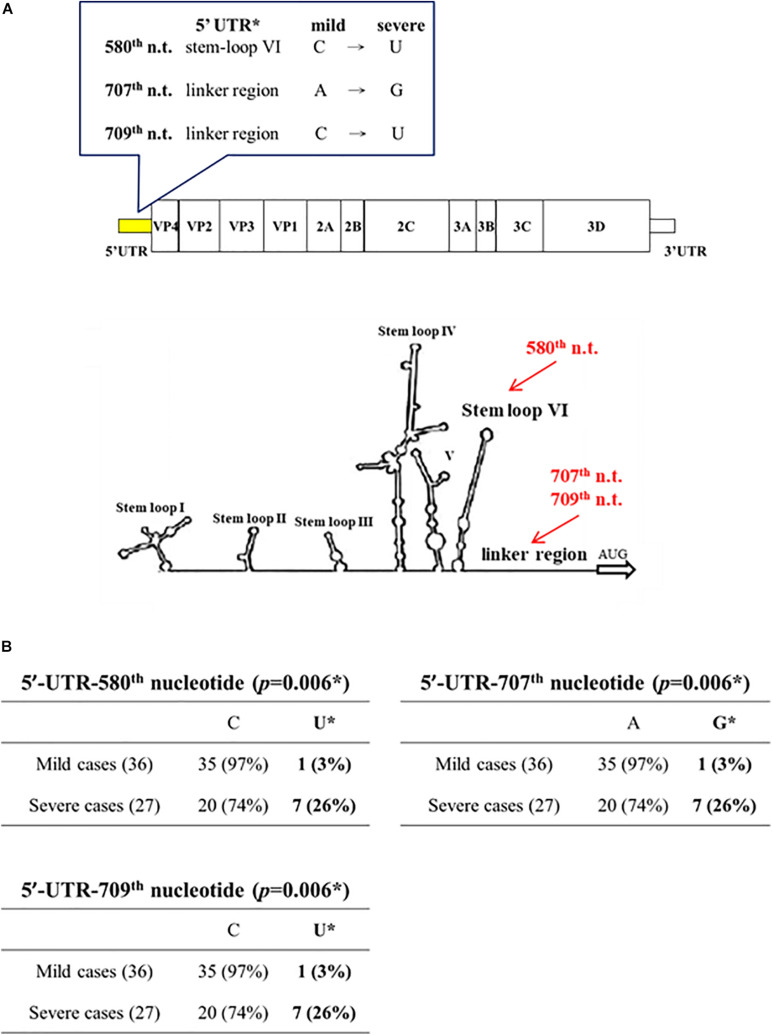
Association of disease severity with three point mutations in 5′ UTR. Genomic sequences of EV-A71 were compared between mild cases (*n* = 36) and severe cases (*n* = 27). **(A)** Three point mutations (red color) were found in the 5′ UTR: C580U, A707G, and C709U. In this 2D diagram of RNA fold, nt 580 is within stem-loop VI, and nt 707 and 709 are in the linker region. **(B)** Statistical analysis of these severity-associated UTR mutations. * *p*-value refers to the Chi-square statistical analysis (χ2 = 7.4574) of the bolded columns. Fisher’s Exact Test (*p* = 0.0166), odds ratio = 12.25 (95% CI = 1.4041∼106.8733). See also Supplementary Figure 1.

By PCR amplification and sequencing, we compared the sequences of the 5′ UTR and the capsid protein VP1 in viral genomes isolated from clinical samples of 36 mild and 27 severe patients. Full-length sequences of viral genomes were also obtained from 7 severe and 8 mild patients. These patients were collected during an EV-A71 outbreak in Taiwan in 2008 (Liao et al., 2014). Except for the 5′ UTR and VP1, we found no mutation associated with disease severity, based on the full-length genome sequences from 7 severe and 8 mild cases. At the 5′ UTR (nt 1 – 747), three mutations C580U, A707G, C709U, were found to occur frequently in severe patients (7/27) (Chi-square *p* = 0.006; Fisher Exact test *p* = 0.0166) (Figure 1 and Supplementary Figure 1). The mutation C580U falls within the stem-loop VI structure, and remains to be investigated whether C580U could affect the cap-independent translation via the internal ribosomal entry site (IRES). Mutations A707G, C709U are located in the linker region between the stem-loop VI and the AUG initiation codon.

Capsid protein VP1 (891 nt) has been used as a standard reference for genotyping. As shown in Figures 2A,B, VP1-145(non-E) is strongly associated with severe cases (26/27, 96%), while VP1-145E is also associated with mild cases (12/36, 33%) (Chi-square *p* = 0.004; Fisher Exact test *p* = 0.0043) (Supplementary Figure 2). These 26 VP1-145(non-E) severe cases include 145Q (16), 145G (9), and 145A (1). Neither 145Q alone nor 145G alone is associated with severe cases with statistical significance.

**FIGURE 2:**
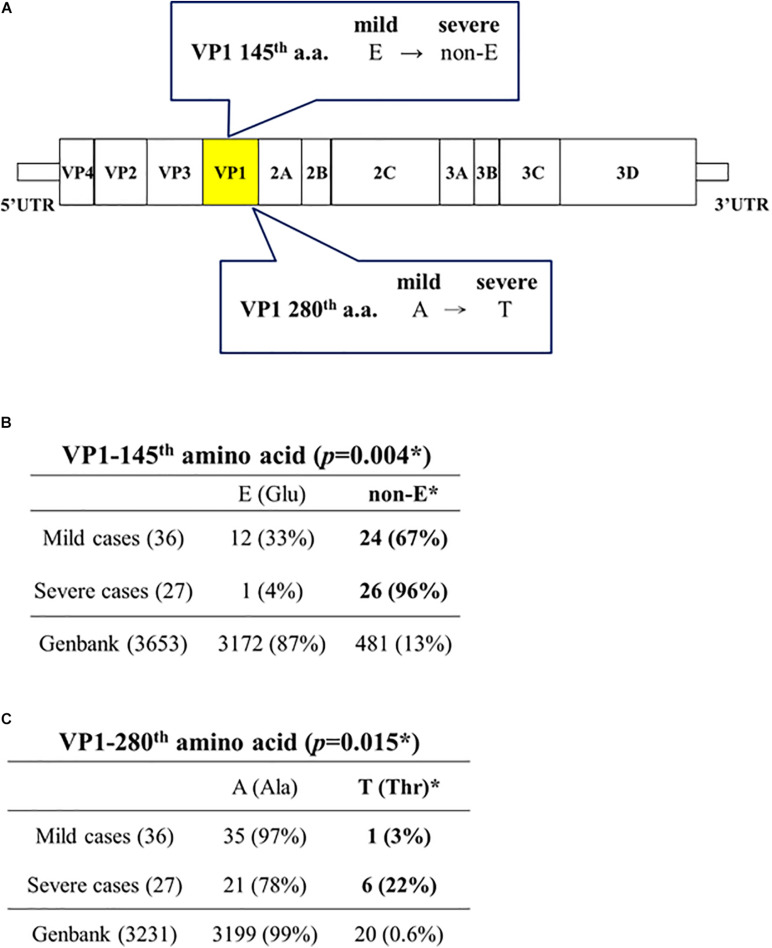
Capsid protein VP1-145(non-E) and VP1-280T are associated with disease severity in EV-A71 infected patients. **(A)** A summary cartoon of VP1 mutations associated with disease severity. **(B)** Statistical analysis revealed a strong association between VP1-145 (non-E) and severe cases. *Chi-square (χ2 = 8.2708) *p* = 0.004; Fisher’s Exact Test (*p* = 0.0043), odds ratio = 13 (95% CI = 1.5697∼107.6669). **(C)** Statistical analysis revealed a significant association between VP1-280T and severe cases. *Chi-square (χ2 = 5.9063) *p* = 0.015; Fisher’s Exact Test (*p* = 0.036), odds ratio = 10 (95% CI = 1.1247∼88.9097). See also Supplementary Figure 2.

GenBank database collected EV-A71 sequences (*n* = 3653, as of April, 2020) deposited at different times from different countries worldwide. For most of these GenBank sequences, no information is available regarding the degree of disease severity of their source patients from the sequence-donating laboratories. Here, we consider these pooled and non-stratified GenBank sequences as a convenient baseline for comparison with our data. It is striking to note that VP1-145(non-E) occurs only at a baseline frequency of 13% (481/3653) in the GenBank database (Figure 2B), including 145Q (256/3653, 7%), 145G (197/3653, 5.4%), 145A (16/3653, 0.4%), and 145R (5/3653, 0.1%). In contrast, VP1-145(non-E) occurs at a 7.4-fold higher frequency (96%, 26/27) in our severe cases. In general, EV-A71 infection caused mainly subclinical or mild symptoms, such as herpangina and HFMD (McMinn, 2002; Shih et al., 2018). Occasionally, it could cause severe disease manifestations, such as neurological disorders, cardiopulmonary failure, and death. The vast majority of VP1 sequences in the GenBank contains a VP1-145E (3172/3653, 87%) (Figure 2B). This fact supports the idea that VP1-145E is preferentially associated with mild cases, and is entirely consistent with our current knowledge about the predominant subclinical or mild symptoms in EV-A71 natural infection. As will be further discussed below, our results of 145E in mild cases and 145(non-E) in severe cases, are exactly opposite to the previous results from animal models (Chua et al., 2008; Huang et al., 2012; Chang et al., 2018; Fujii et al., 2018; Kobayashi et al., 2018; Tee et al., 2019).

Unexpectedly, among those 7 severe cases containing 5′ UTR mutations, 6 out of 7 cases contain a novel alanine-to-threonine mutation at the VP1 position 280 (A280T) (Figure 2A). We found 99% (3199/3231) of VP1 sequences in the GenBank contains a VP1-280A, and only 0.6% contains a VP1-280T (20/3231). In contrast, 22% (6/27) in our severe cases contains a VP1-280T (Figure 2C and Supplementary Figure 2). This frequency of occurrence (22%) is near 37-fold higher than the baseline frequency (0.6%) of the GenBank database.

**FIGURE 3:**
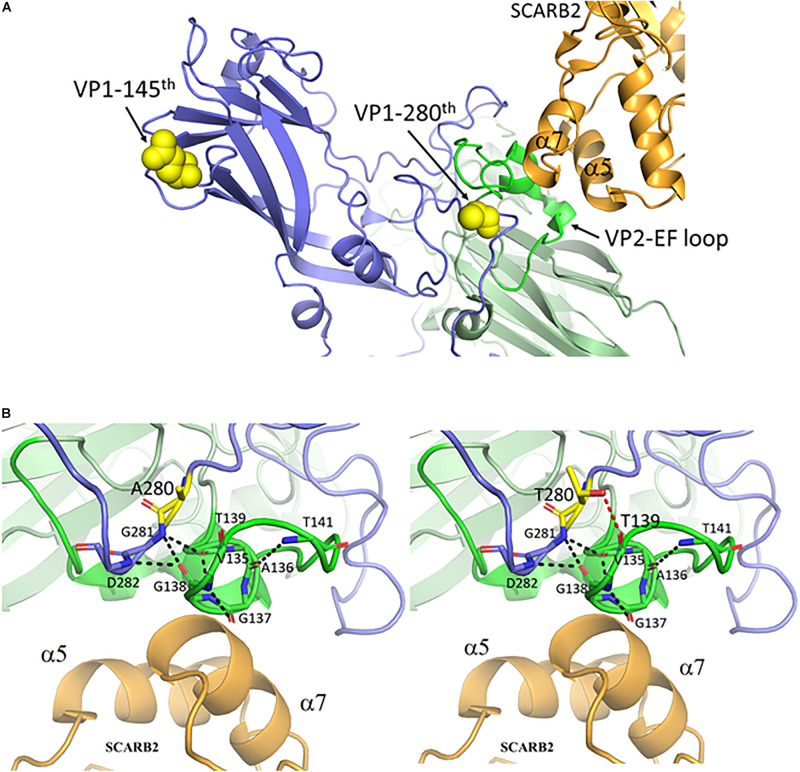
The mutant threonine of VP1-280T is located at the interaction hub between VP1, VP2 and the entry receptor hSCARB2. **(A)** VP1 amino acid 280, but not amino acid 145, is near the contact site between EV-A71 and hSCARB2, according to the cryoEM structure of the binding complex of VP1, VP2 and hSCARB2 at low pH (Zhou et al., 2019). PDB accession code: 6I2K. Orange, human SCARB2; Blue, VP1 capsid protein; Green, VP2-EF loop; Yellow, VP1 amino acids 145 and 280. **(B)** A loop turn of the VP2-EF loop (green) is in contact with the α7 helix of SCARB2 (orange). The backbone atoms of VP1 and the VP2 EF loop are represented as sticks with carbon atoms colored in light blue and green, respectively. This VP2 loop turn can be stabilized by a hydrogen-bonded network (black dashed lines) between the backbone oxygen and nitrogen atoms of VP1 G281-D282 and those of VP2 V135-T141. One *de novo* created hydrogen bond between the oxygen atom (hydroxyl group) of the mutant VP1-280T and the carbonyl group of VP2-139T is colored in a red dashed line. Residues A280 and T280 of VP1 are shown in sticks with carbon atoms in yellow. This figure is produced using Discovery Studio Visualizer and PyMOL (see section “Materials and Methods”).

EV-A71 can bind to SCARB2 or PSGL-1 (P-selectin glycoprotein ligand-1) receptors for entry (Nishimura et al., 2009; Yamayoshi et al., 2009). The structure of the binding complex of EV-A71 and SCARB2 had recently been resolved by cryoEM at 3.4 - angstron resolution (Zhou et al., 2019). Coincidentally, amino acid 280 of VP1 is located in the immediate neighborhood of the contact site between VP2 and the SCARB2 receptor (Figure 3A). A close-up view at the contact site in Figure 3B revealed the interaction between a VP2 loop turn (green) and the α7 helix of SCARB2 (orange). This loop turn of VP2 may be stabilized by a web-like interaction structure, which consists of multiple hydrogen bonds (black dashed lines) between VP1-281G, VP1-282D (blue), and VP2 135V – 141T (green). The VP1 mutation from alanine-to-threonine at amino acid 280 could generate an extra H-bond between VP1-280T and VP2-139T (red dashed line), which in turn could enhance the interaction between the virus and its SCARB2 entry receptor.

**FIGURE 4:**
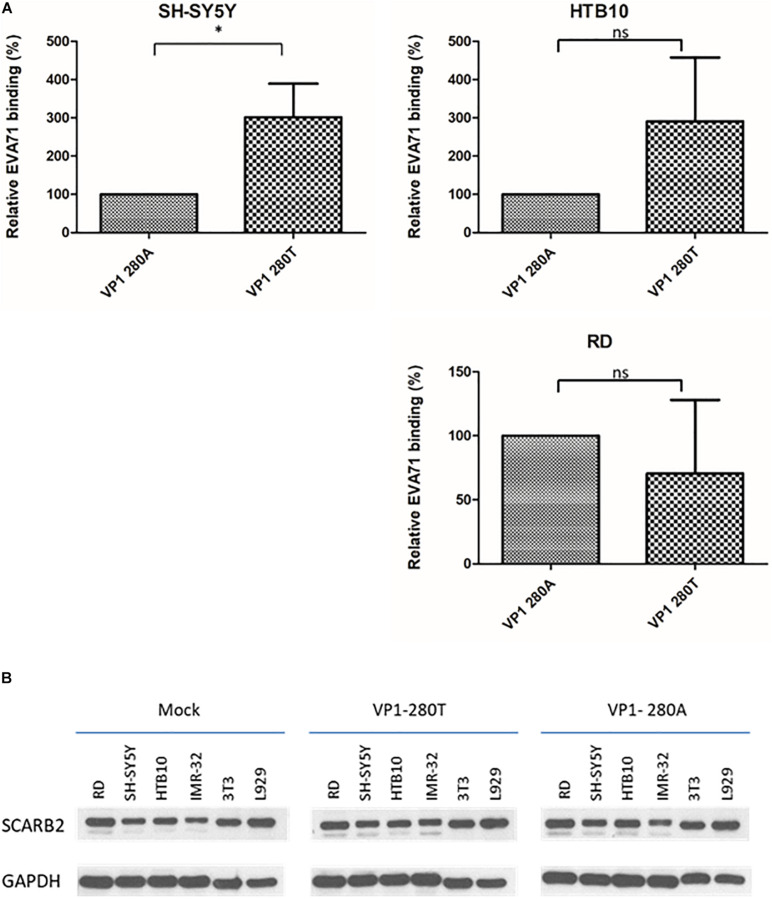
EV-A71 enterovirus VP1-280T bound to a human neuroblastoma cell line SH-SY5Y better than the other virus VP1-280A. **(A)** The binding assay was performed as described in section “Materials and Methods.” SH-SY5Y and HTB10: human neuroblastoma cell lines, RD: a human rhabdomyosarcoma cell line. *Student’s *t*-test statistical analysis *p* < 0.05. **(B)** Comparisons of the SCARB2 expression levels among different cells infected with viruses VP1-280T vs. VP1-280A. Western blot analysis was performed for the detection of SCARB2 proteins using an anti-SCARB2 antibody. IMR32: human neuroblastoma cell line, 3T3 and L929 are mouse fibroblast cell lines. Mock, uninfected culture; GAPDH, an internal control.

We compared the binding activities between VP1-280A and VP1-280T viruses with human rhabdomyosarcoma cell line RD, and human neuroblastoma cell lines HTB10 and SH-SY5Y (Figure 4A). No difference in their respective binding activities was detected between VP1-280A and its congenic (isogenic) VP1-280T using HTB10 or RD cells. However, VP1-280T appeared to bind better to SH-SY5Y cells than VP1-280A. The expression levels of SCARB2 were similar in various host cell lines infected with VP1-280T or VP1-280A by Western blot analysis (Figure 4B). Therefore, it remains to be investigated why SH-SY5Y binds better with VP1-280T, rather than VP1-280A. We cannot exclude the possibility that SCARB2 polymorphism may exist between these cell lines tested for binding.

## Discussion

In literature, at least 10 mutations at the 5′ UTR have been associated with disease severity, including nt 150, 158, 241, 272, 488, 494, 571, 579, 606, and 700 (Table 1). None of these mutations in previous reports overlapped with our current mutations at nt 580, 707, and 709. The 5′ UTR is known to contain *cis-*elements important for both IRES-mediated cap-independent translation and the replication origin for genome multiplication. While nucleotide 580 falls within the stem-loop VI and IRES (Figure 1), nt 707 and 709 are in the linker region between IRES and the first AUG initiation codon. To investigate whether these novel 5′ UTR mutations have any functional significance in IRES-mediated translation, we conducted reporter assays in human RD cells (data not shown). Although we observed no significant difference in the reporter activities between the WT and mutant 5′ UTR, it remains possible that these mutations could play a role in genome replication in viral life cycle.

In addition to 5′ UTR, severity-associated mutations had been reported to occur at VP1, VP2, 2A, and 3D (Table 1). It should be noted that some mutations, marked with a ^∗^ symbol in Table 1, were evaluated in mouse models. Therefore, they could reflect mouse-specific adaptive mutations, rather than *bona fide* naturally occurring mutations in human patients. For example, VP1-145E has been shown to contribute to viremia, lethality or pathogenesis in animal models (Chua et al., 2008; Huang et al., 2012; Kataoka et al., 2015; Chang et al., 2018; Fujii et al., 2018; Kobayashi et al., 2018; Tee et al., 2019). However, in clinical samples, VP1-145E does not appear to be associated with severe cases (Figure 2B and further discussions below) (Li et al., 2011).

For example, a viral strain MP-26M from Australia was generated *de novo* by serial passages through newborn BALB/c mice (Chua et al., 2008). It harbored an artificial VP1-G145E adaptive mutation with increased virulence in mice. In another mouse-passaged strain MP4, it contained a mouse-adapted mutation VP1-Q145E. This VP1 Q145E mutation is absent in the parental clinical isolate from a Taiwanese patient, and exhibited increased infectivity *in vitro* and lethality in ICR mice (Huang et al., 2012). Conversely, consistent with these earlier reports, a viral strain EV-R containing a VP1-E145G mutation was found recently to become less virulent in hSCARB2 transgenic mice (Chang et al., 2018). Most recent studies from Japan also demonstrated that VP1-145E is associated with neurovirulence in suckling mice, hSCARB2 transgenic mice, and cynomolgus monkeys (Fujii et al., 2018; Kobayashi et al., 2018). Taken together, VP1-145E has always been shown to be a virulence determinant in mice. Unexpectedly, in our human samples, it is VP1-145(non-E) that is almost exclusively associated with human severe cases (26/27, 96%) (Figure 2B). Furthermore, in GenBank database, 87% (3172/3653) of the sequences contain a VP1-145E. In natural infection with EV-A71, mild symptoms are the most common clinical outcome. For example, in the 1998 epidemic in Taiwan, case severity rate is merely around 0.3% (405/129, 106) (Ho et al., 1999). In China, between 2008 and 2012, a reported case severity rate is only around 1.1% (Xing et al., 2014). Therefore, it is reasonable to believe that the vast majority of EV-A71 sequences (87%) deposited in the GenBank, represents mainly virus isolates from the most common mild cases (99%), rather than from the extremely rare severe cases (0.3 – 1.1%).

Is it possible that this discrepancy between human and mouse studies in this VP1-145E related virulence, is actually related to the EV-A71 genotypic difference? A previous report found no correlation between genotypes and virulence (Li et al., 2011). As summarized in Table 1, the viruses used in human VP1-145 studies are of the genotypes B5 and C4 (Li et al., 2011; Chang et al., 2012), while the viruses used in mouse model studies are of genotypes C2, B2, B3, and B4 (Table 1; Chua et al., 2008; Huang et al., 2012; Chang et al., 2018; Fujii et al., 2018; Kobayashi et al., 2018). No common genotype has ever been used in both human and mouse studies. We consider it most likely that 145E is artificially selected from the viral adaptation in the mouse environment.

It is noteworthy that in our mild cases, 67% (24/36) also contained a 145(non-E) (Figure 2B). It is quite possible that VP1-145(non-E), by itself, is not sufficient, or not the only major determinant for *in vivo* virulence. Viral mutations other than VP1-145(non-E), such as VP1-280T, could also contribute to the pathogenesis. The potential linkage relationship of these severity-associated mutations is shown in Table 2. Among the 7 severe patients containing 5′ UTR mutations, 6 out of 7 severe cases also contain a VP1-A280T mutation. Finally, individual’s age and host immunity are also major factors in pathogenesis (Liao et al., 2014; Liou et al., 2016, 2019; Shih et al., 2018). It cannot be excluded that some of the borderline severe cases (in the gray zone) may have been classified into the category of mild cases.

**TABLE 2:** Linkage of EV-A71 virulent mutations in 27 severe cases.

	**5′-UTR nucleotide #**			**VP1 amino acid #**	
	**580**	**707**	**709**	**145**	**280**
No. F1	U	G	U	Gly	Thr
No. F6	U	G	U	Gly	Ala
No. F7	U	G	U	Gly	Thr
No. F9	U	G	U	Gln	Thr
No. F17	U	G	U	Glu	Thr
No. F22	U	G	U	Gln	Thr
No. F23	U	G	U	Gln	Thr
No. 1	C	A	C	Gln	Ala
No. 5	C	A	C	Gln	Ala
No. 6	C	A	C	Gln	Ala
No. 26	C	A	C	Gly	Ala
No. F2	C	A	C	Gly	Ala
No. F3	C	A	C	Gln	Ala
No. F4	C	A	C	Gln	Ala
No. F5	C	A	C	Gln	Ala
No. F8	C	A	C	Gly	Ala
No. F10	C	A	C	Gln	Ala
No. F11	C	A	C	Gln	Ala
No. F12	C	A	C	Gly	Ala
No. F13	C	A	C	Gln	Ala
No. F14	C	A	C	Gln	Ala
No. F15	C	A	C	Gln	Ala
No. F16	C	A	C	Gln	Ala
No. F18	C	A	C	Gly	Ala
No. F19	C	A	C	Gln	Ala
No. F20	C	A	C	Gly	Ala
No. F21	C	A	C	Ala	Ala

VP1 amino acid 145 has been considered as a key residue for binding to heparan sulfate and PSGL-1 receptor (Nishimura et al., 2013; Tan et al., 2017; Fujii et al., 2018; Tee et al., 2019; Kobayashi et al., 2020). While strain VP1-145G/Q can bind to PSGL-1, the viral strain VP1-145E cannot bind to PSGL-1 (Nishimura et al., 2013). Relative to the VP1-145Q virus, VP1-145E virus is a weak heparan-binder, yet correlated with increased virulence in animal models (Fujii et al., 2018; Kobayashi et al., 2018). It was therefore hypothesized that the VP1-145Q virus could be attenuated in mice by its better binding to heparan sulfate of the peripheral tissues (Fujii et al., 2018; Kobayashi et al., 2018). Heparan sulfate on the cell surface is a very common attachment site for many other viruses, including herpesvirus, human immunodeficiency virus and hepatitis B virus (Shukla and Spear, 2001; Connell and Lortat-Jacob, 2013; Choijilsuren et al., 2017). It would be interesting to ask whether poor heparin-binding viruses other than EV-A71 can also predict virulence.

Using a RD-A cell line, Kobayashi et al. (2020) reported rapid and frequent selection and adaptation of heparan sulfate-binding variants, changing from VP1-145E to VP1-145Q/G. Is it possible that the tight association (26/27, 96% in Figure 2B) between disease severity and VP1-145(non-E), simply reflects a bias in cell culture? It will be ideal if one can compare the VP1 sequences before and after passages through the cell culture. However, in most cases, the amount of clinical samples are scarce, and not available for direct PCR amplification and sequencing. Overall, we consider it highly unlikely for the following reasons: *First*, we used the same RD cells to amplify EV-A71 from both severe (*n* = 27) and mild cases (*n* = 36). There is no reason that the same RD cells preferentially select for 145(non-E) variant, only when the virus is from severe cases, but not when the virus is from mild cases. Why should the RD cells discriminate between viruses originating from severe versus mild cases? *Second*, the phenomenon observed in the RD-A cell line (Kobayashi et al., 2020) may not necessarily be applicable to the RD cell line. Presumably, this RD-A cell line is a derivative of the RD cell line. *Third*, there are more than 3,000 entries of VP1 sequences in GenBank database. The vast majority (87%) contained VP1-145E (Figure 2B). In epidemiological studies, 99% of EV-A71 infected children are mild cases (Ho et al., 1999; Xing et al., 2014). In all studies on EV-A71 reported to date, the RD cell line, instead of the RD-A cell line, was always the most commonly used in literature (Table 1). It would be most informative in the future if RD and RD-A cell lines will be compared side-by-side in the same experimental setting in the future.

In GenBank database, there are 20 EV-A71 sequences containing a VP1-280T mutation (Supplementary Table 1). Further examination of these 280T variant sequences revealed that most of them are of genotype B5 from the 2008 epidemic in Taiwan, albeit two sequences are of genotype C1 from United Kingdom, and one is of genotype C4 from China. Therefore, while mutation VP1-280T is associated with disease severity of patients infected with genotype B5 (Figure 2C), it is likely that this 280T mutation may be associated with severe cases infected with other genotypes, such as C1 and C4.

Recently, the initial attachment complex between EV-A71 and SCARB2 at low pH was visualized by cryoEM (Zhou et al., 2019). It reveals that SCARB2 binds EV-A71 on the southern rim of the canyon, rather than across the canyon as predicted previously (Chen et al., 2012; Dang et al., 2014; Zhang et al., 2017). This new structure suggests an allosteric mechanism for receptor binding, and the subsequent uncoating of EV-A71 in the low-pH lumen of endosome or lysosome. According to this complex structure, VP1-145 is far away from the contact site between SCARB2 and VP2 (Figure 3A). Interestingly, we found VP1-280 is in close proximity to SCARB2 and VP2 (Figure 3B). We entertain the possibility that VP1-280T could form hydrogen bonding with VP2-139T and help stabilize the VP2 interaction with SCARB2. This ternary complex could play an important role in the viral attachment and entry into the host cells. We found no consensus sequences for serine/threonine-kinase substrates around VP1-280T and VP2-139T. O-glycosylation at these residues cannot be excluded.

While VP1-145E does not bind to PSGL-1 receptor, VP1-145Q/G was shown to bind to PSGL-1 (Nishimura et al., 2013). Unlike VP1-280, VP1-145 is far away from the SCARB2 binding site (Figure 3A). Taken together, we report here that VP1-145(non-E) could be associated with an increased risk for disease severity, most likely via the PSGL-1 receptor-mediated entry pathway. In contrast, VP1-280T could contribute to virulence via a *de novo* created hydrogen bonding with the highly conserved VP2-139T. This novel hydrogen bonding could help stabilize a VP2 loop turn by strengthening a web-like interaction structure of the VP1-VP2 complex, leading to more efficient infection via the SCARB2 receptor-mediated entry pathway. These severity-associated mutations might be useful for patient care as a diagnostic tool in predicting the course of disease progression.

## Data Availability Statement

The datasets presented in this study can be found in online repositories. The names of the repository and accession number(s) can be found below: https://www.ncbi.nlm.nih.gov/genbank/, accession numbers (MT360921-360998 and MT348284-348346).

## Author Contributions

C-SC, C-CL, and CS: experimental design. Y-YY, C-YL, and J-SL: virus isolates. C-SC and C-CL: conduct the experiments. C-SS, M-JH, C-SC, and CS: bioinformatics. CS and C-SC: writing. All authors: data analysis.

## Conflict of Interest

The authors declare that the research was conducted in the absence of any commercial or financial relationships that could be construed as a potential conflict of interest.
