# Supplementary material for: Novel Naturally Occurring Mutations of Enterovirus 71 Associated With Disease Severity
Source: Front Microbiol. 2021 Jan 13;11:610568. doi: 10.3389/fmicb.2020.610568 (PMC7838335; doi:10.3389/fmicb.2020.610568)
Supplement: Supplementary file 1 [file Data_Sheet_1.PDF]

Supplementary Material

Title: Novel Naturally Occurring Mutations of Enterovirus 71 Associated with Disease Severity

Short title: EV-A71 virulence determinants and mutations

Authors: [1, 2]Chih-Shin Chang, [2]Chun-Che Liao, [2,3,4]An-Ting Liou, [2]Yi-Chun Chou, [5]Ya-Yen Yu, [5]Chi-Yung Lin, [5]Jen-Shiou Lin, [2]Ching-Shu Suen, [2]Ming-Jing Hwang, and [2,3]Chiaho Shih*

[1] Genomics Research Center, Academia Sinica, Taipei, Taiwan

[2] Institute of Biomedical Sciences, Academia Sinica, Taipei, Taiwan

[3] Graduate Institute of Medicine, Kaohsiung Medical University, Kaohsiung, Taiwan

[4]Drug Development and Value Creation Research Center, Kaohsiung Medical University, Kaohsiung, Taiwan

[5] Section of Clinical Virology and Molecular Diagnosis, Department of Laboratory Medicine, Changhua Christian Hospital, Changhua, Taiwan

Supplementary Figure 1.

Mild cases 5'-UTR-580th nucleotide

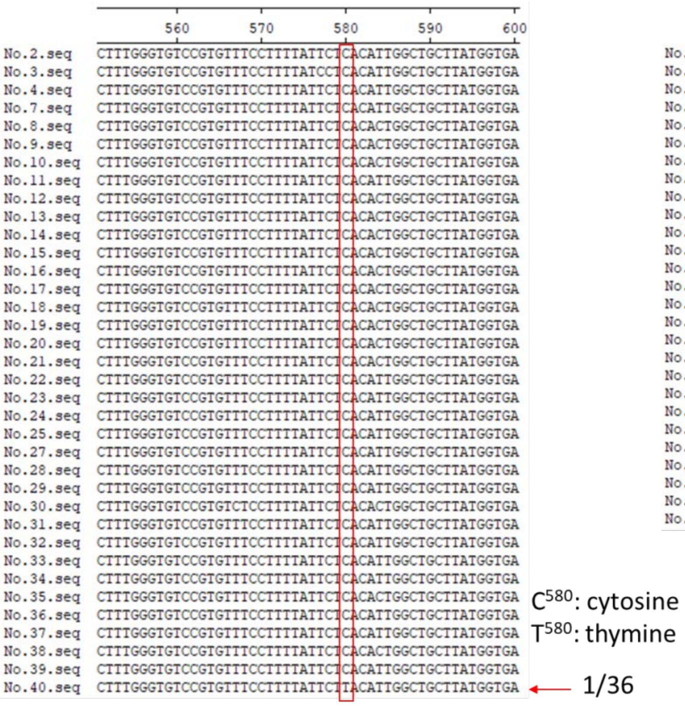

Severe cases 5'-UTR-580th nucleotide

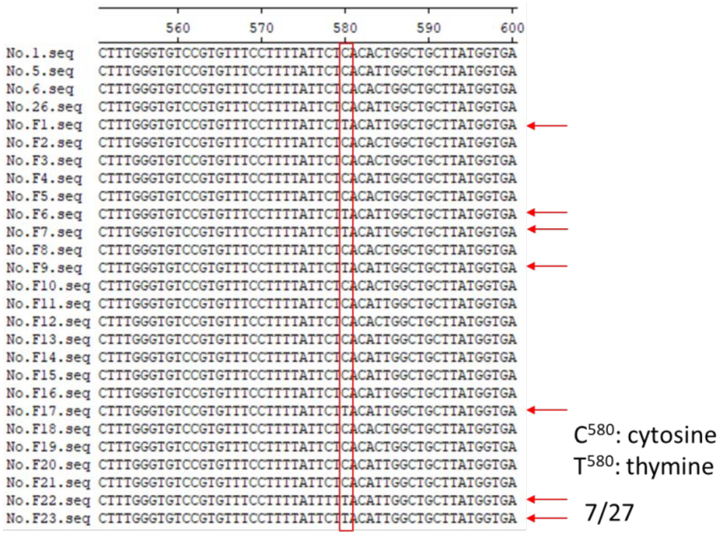

Mild cases 5'-UTR-707th & 709th nucleotide

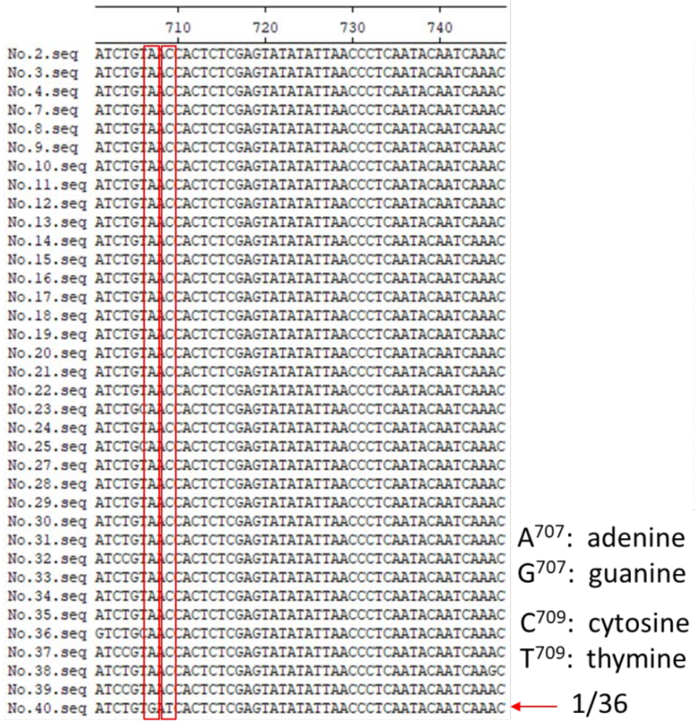

Severe cases 5'-UTR-707th & 709th nucleotide

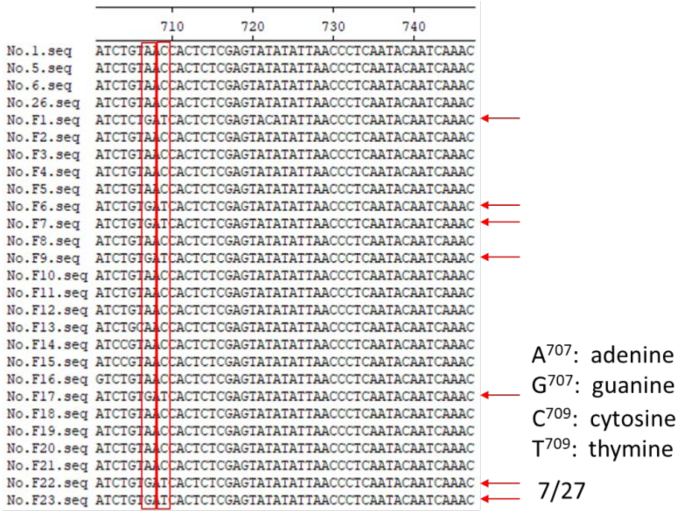

Supplementary Figure 1. Three 5' UTR mutations were found to be associated with severe diseases by multiple sequence alignment. *Upper panel*: Mutation C580T is present in 7 out of 27 severe cases, and only 1 out of 36 mild cases. *Lower panel*: Both mutations A707G and C709T are present in 7 out of 27 severe cases, and only 1 out of 36 mild cases.

Supplementary Figure 2.

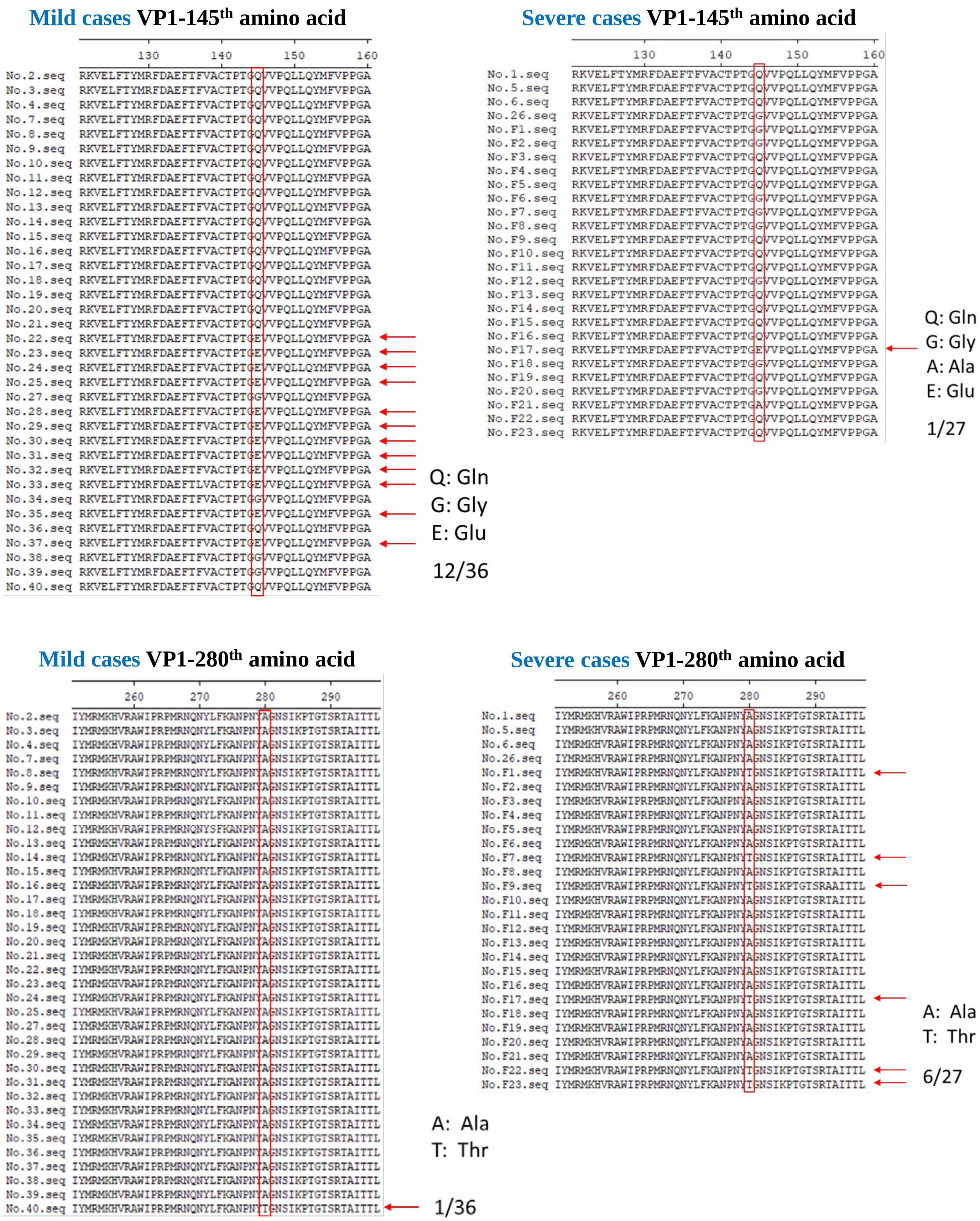

Supplementary Figure 2. Two VP1 mutations were found to be associated with severe diseases by multiple sequence alignment. *Upper panel*: VP1 mutation 145E is present in 12 out of 36 mild cases, and only 1 out of 27 severe cases. *Lower panel*: VP1 mutation 280T is present in 6 out of 27 severe cases, and only 1 out of 36 mild cases.

Supplementary Figure 3.

A

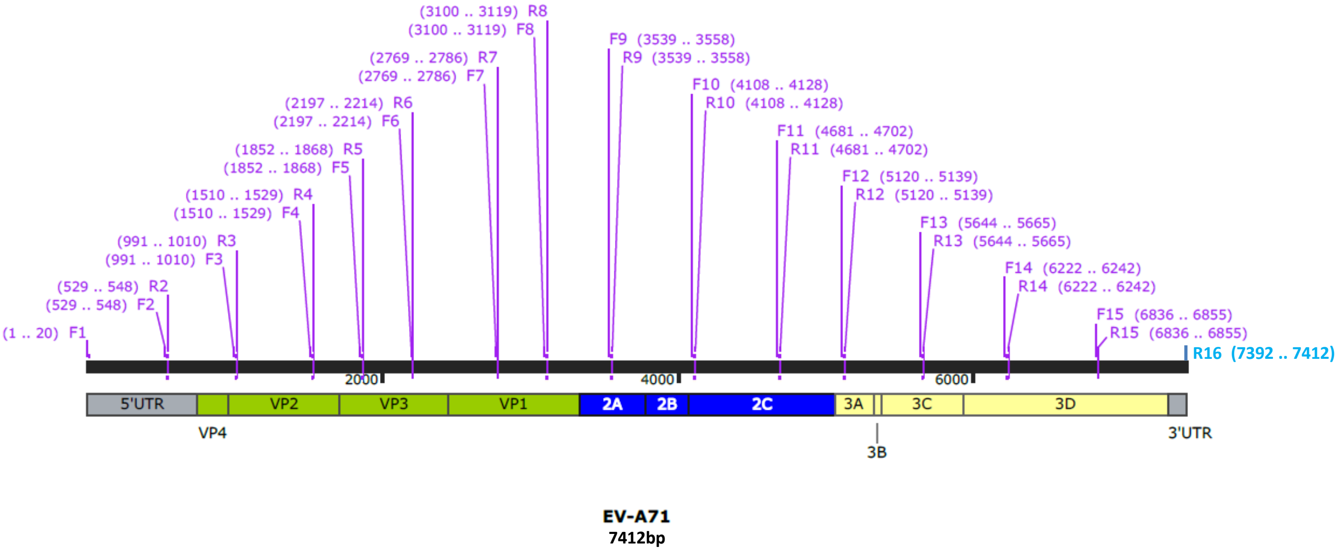

B

Name	Sequence
F1	TTAAAACAGCCTGTGGGT
F2	AACTCTGCAGCGGAACCGAC
R2	GTCGGTTCCGCTGCAGAGTT
F3	GTGGCACAACTCACCATTGG
R3	CCAATGGTGAGTTGTGCCAC
F4	CGGACCAATAACTGTGCCAC
R4	GTGGCACAGTTATTGGTCCG
F5	TTGTGTCAAGTGGAGAC
R5	GTCTCCACTTGACACAA
F6	CTACAATCATCTGTCACC
R6	GGTGACAGATGATTGTAG
F7	GGATATAGACATAACTGG
R7	CCAGTTATGTCTATATCC
F8	CTTGAATATGGAGCGTGTCC
R8	GGACACGCTCCATATTCAAG
F9	GAAAGCACTATCCAGTCAGC
R9	GCTGACTGGATAGTGCTTTC
F10	GATATGGCGAGTGCCGCCAAG
R10	CTTGGCGGCACTCGCCATATC
F11	CCACCAATGGCTTCTCTTGAAG
R11	CTTCAAGAGAAGCCATTGGTGG
F12	CAGACGCTATTAGCGATCTC
R12	GAGATCGCTAATAGCGTCTG
F13	GATATCACCAAGTTCATTCCAG
R13	CTGGAATGAACTTGGTGATATC
F14	GATGAGCATGGAGGAAGCGTG
R14	CACGCTTCCTCCATGCTCATC
F15	CAATGATCAACAACATCATC
R15	GATGATGTTGTTGATCATTG
R16	GCTATTCTGGTTATAACAAAT

Supplementary Figure 3 . A sequencing strategy of the full-length EV-A71 viral genomes. A) Cartoom illustration of the locations of sequencing primers. **B)** Nucleotide sequences of the sequencing primers.

Table S1 Twenty EV-A71 viruses in GenBank containing a VP1-A280T mutation

Accession No.	Country	Virus genotype	Disease severity	Ref.
CAP74024	United Kingdom	C1	Tonsilitis**	Bible et al., 2008
CAP74025	United Kingdom	C1	Vomiting and diarrhea**	Bible et al., 2008
AEE00789	China	C4	N.A.	Liu et al., 2010*
APM86731	Russia	N.A.	N.A.	Sevostianova et al., 2010*
QBP14904	Vietnam	B5	N.A.	Hoang., 2018*
ADK88905	Taiwan	B5	N.A.	Hsieh et al., 2010*
AGT28543	Taiwan	B5	N.A.	Wu et al., 2013
AGT28626	Taiwan	B5	N.A.	Wu et al., 2013
AGT28627	Taiwan	B5	N.A.	Wu et al., 2013
AGT28637	Taiwan	B5	N.A.	Wu et al., 2013
AGT28670	Taiwan	B5	N.A.	Wu et al., 2013
AGT28673	Taiwan	B5	N.A.	Wu et al., 2013
AHC99280	Taiwan	B5	N.A.	Huang et al., 2013
AHC99285	Taiwan	B5	N.A.	Huang et al., 2013
AHC99286	Taiwan	B5	N.A.	Huang et al., 2013
AYP73828	Taiwan	B5	N.A.	Lee et al., 2018
ADE80716	Taiwan	B5	HFMD**	Chang et al., 2012
ADB97998	Taiwan	B5	N.A.	Lee et al., 2010
ADD52076	Taiwan	B5	N.A.	Lee et al., 2010
ADD52075	Taiwan	B5	N.A.	Lee et al., 2010

* GenBank deposited sequences. **These virus strains were likely to be isolated from mild cases. NA: No information is available about the patient’ disease severity. HFMD, hand, foot, and mouth disease.
